# A Rare Complication of Cardiac Ablation: Atrial-esophageal Fistula Presenting as Odynophagia

**DOI:** 10.7759/cureus.6871

**Published:** 2020-02-04

**Authors:** Muzammil Khan, Mamoon Ur Rashid, Hammad Zafar, Waqas Ullah, Abu H Khan

**Affiliations:** 1 Internal Medicine, Khyber Teaching Hospital, Peshawar, PAK; 2 Internal Medicine, AdventHealth, Orlando, USA; 3 Internal Medicine, Advent Health, Orlando, USA; 4 Internal Medicine, Abington Hospital-Jefferson Health, Abington, USA; 5 Gastroenterology, AdventHealth, Orlando, USA

**Keywords:** atrial esophageal fistula, atrial fibrillation, cardiac ablation, chest pain, odynophagia

## Abstract

Radiofrequency catheter ablation has been commonly used for the treatment of drug-refractory atrial fibrillation. The esophageal injury along with the development of atrial-esophageal fistula (AE fistula) is fairly rare but is a devastating complication of catheter ablation. Described in 2004 for the first time, it is the most lethal of all the complications of catheter ablation with a high mortality rate. The clinical presentation of an AE fistula is variable, however, early diagnosis and treatment can prevent a fatality. We have reported a case of an AE fistula post catheter ablation for drug-resistant atrial fibrillation, along with its treatment, diagnosis, and possible preventive measures.

## Introduction

An atrial-esophageal (AE) fistula can be defined as an abnormal connection between the esophagus and the left atrium of the heart. The AE fistula is a rare but serious complication post catheter ablation for drug-refractory atrial fibrillation. The most common strategy applied is creating circumferential lesions around the pulmonary vein ostia with or without ablation lesions within the left atrium. The incidence of AE fistulas post-ablation has been reported to be around 0.015% to 0.04%, however, this may be underreported, as it remains undiagnosed in most patients [1]. AE fistulas have been associated with a mortality rate of 40%-100% [1-2]. A lack of clinical awareness and delayed diagnosis has been the major cause of the high mortality rate of AE fistulas post-ablation [2]. We report a rare and grave complication of catheter ablation procedure for resistant atrial fibrillation presenting with esophageal injury and, subsequently, an AE fistula (the abstract of this case was published in the American College of Gastroenterology in October 2019) [3].

## Case presentation

**Figure 1:**
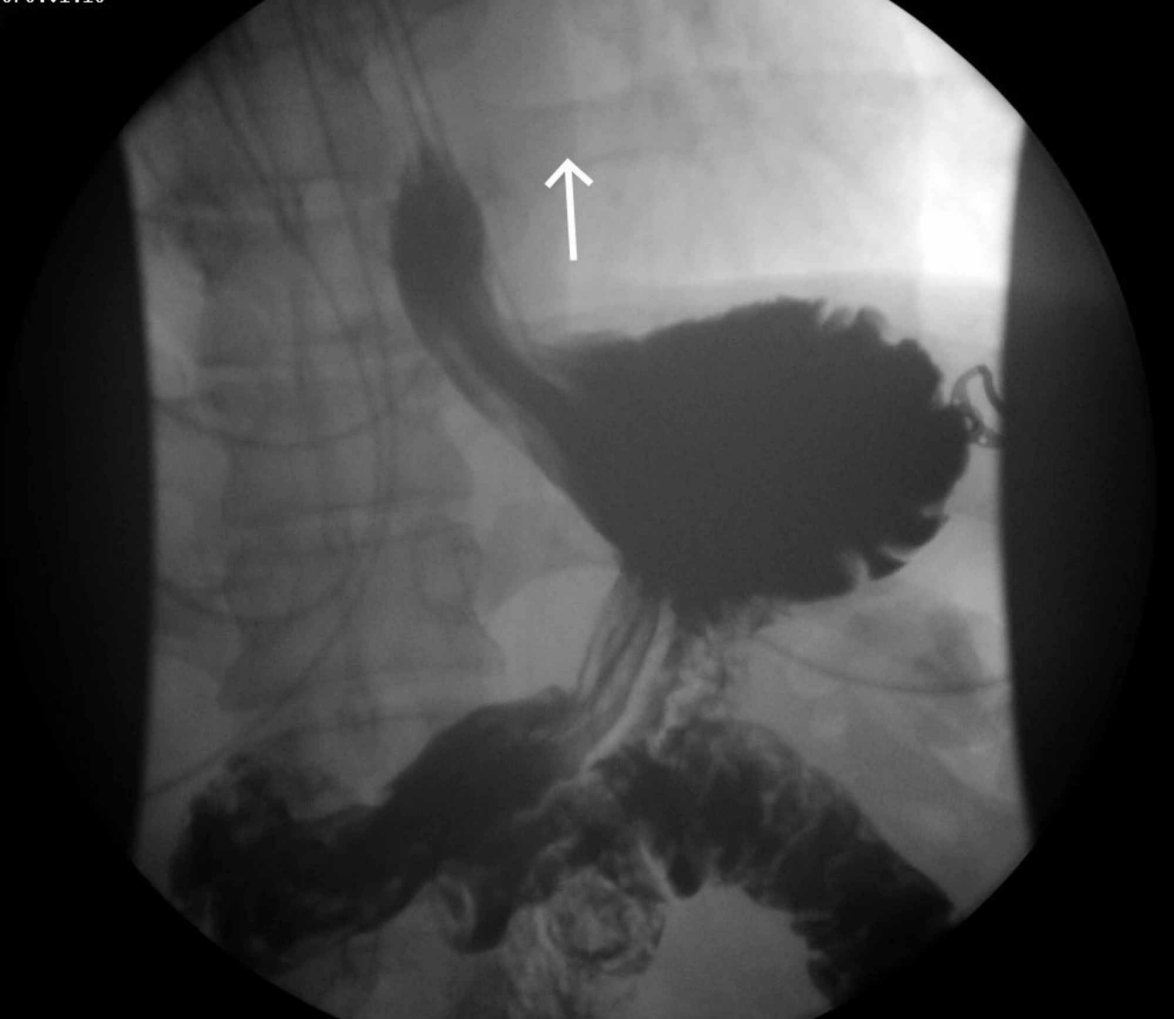
Upper GI series demonstrating an esophageal tear (arrow) GI: gastrointestinal

**Figure 2:**
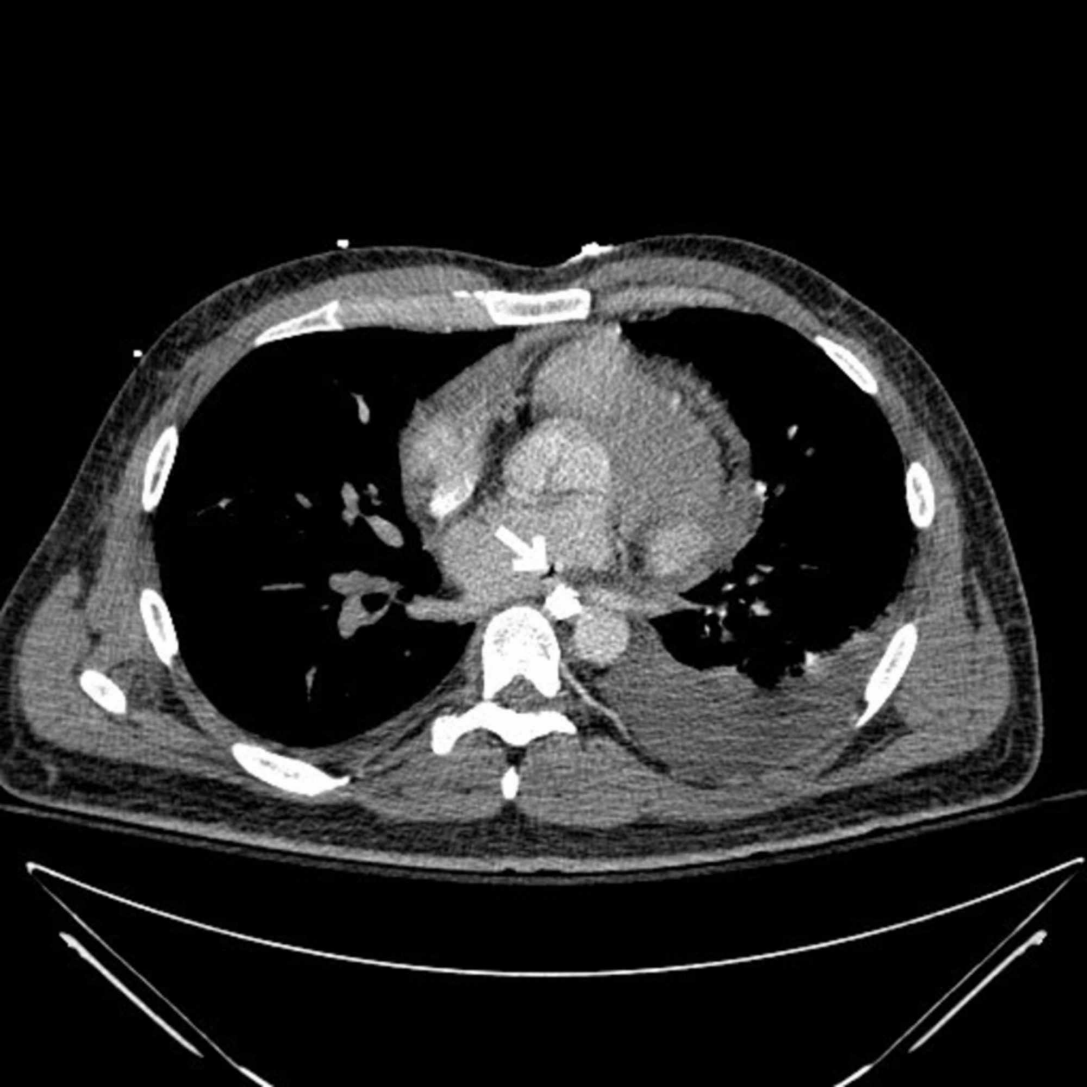
Chest CT (pulmonary veins) showing an atrial-esophageal fistula (arrowhead) CT: computed tomography

A 43-year-old male presented to the emergency department (ED) with complaints of retrosternal chest pain and odynophagia. He had a history of atrial fibrillation (AF) s/p cardiac ablation four days ago. The patient had a total of four cardioversions in the past for AF and was also treated with both antiarrhythmics and rate control medications, but his symptoms persisted during hospitalization. The initial investigation, including troponins, chest X-ray, and electrocardiogram (EKG), was normal. Computed tomography (CT) chest with contrast was specifically done to rule out complications related to cardiac ablation, which was unremarkable. The patient then underwent an upper gastrointestinal (GI) series with gastrografin, a water-soluble contrast medium, which showed a focal contrast collection at the distal esophagus at the level of the heart (Figure 1). This was concerning for esophageal injury, one of the known complications of cardiac ablation, status post recent atrial fibrillation ablation. The GI team decided to go for esophagogastroduodenoscopy (EGD), and it was seen that the patient had a linear, deep, mid-esophageal perforation for which six hemostatic clips were used for closure. The patient was later discharged on parenteral nutrition (TPN). After two weeks, the patient presented again with complaints of shortness of breath and chest discomfort. Examination revealed loud pericardial friction rub suspicious for pericarditis and cardiac tamponade. Stat Echo was done, which showed pericardial effusion (2.5 cm) and early signs of cardiac tamponade. Emergent pericardiocentesis was performed and a pericardial drain was placed. After pericardiocentesis, the patient became febrile. Workup for sepsis was initiated, including blood cultures. Anaerobic species, Lactobacillus, were growing in blood cultures. CT chest (pulmonary veins) was ordered for further investigation and an AE fistula (Figure 2) was found with left-sided pleural effusion, suspected for infection. The cardiothoracic surgery team was consulted and surgery was planned. The patient underwent left thoracotomy, decortication of empyema, and repair of the inferior pulmonary vein/atrial margin, esophageal repair, gastrostomy-jejunostomy (G-J) tube placements, and omental transfer. The patient eventually improved and was discharged to rehab.

## Discussion

Atrial fibrillation is the most common and clinically significant cardiac arrhythmia in the United States. While pharmacologic therapy remains the cornerstone of treatment for atrial fibrillation, percutaneous catheter ablation of the left atrium and pulmonary vein ostia has become a successful modality in patients with drug-refractory atrial fibrillation. Radiofrequency ablation is performed by creating circumferential lesions in the left atrium or pulmonary vein ostia. Historically, an AE fistula was reported as an intraoperative complication of cardiac surgery. The first case reported outside of the operating room was back in 2004.

However, due to the increased use of catheter ablation for the treatment of drug-refractory atrial fibrillation, there has been an associated risk of complications as well. Of the most commonly reported are cardiac tamponade, AE fistula, stroke, venous thromboembolism, myocardial infarction, pneumonia, and sepsis. The second most common cause within 30 days of the procedure was an atrioesophageal fistula, the most common being cardiac tamponade [4].

The pathophysiology of AE fistulas is dependent on the relation between the anatomic location of the esophagus and the left atrium or pulmonary vein ostia. The esophagus lies close to the left atrium. It grooves behind the left atrial wall and overlapping zone of the pulmonary vein ostium where it is highly susceptible to injury due to thermal energy provided by radiofrequency ablation. The posterior wall of the left atrium has non-uniform thickness with thinnest superiorly where the esophagus passes and thickest adjacent to the coronary sinus. The varying presentation of fibrofatty tissue present between the left atrium and the esophagus also plays a role in the development of esophageal injury [5]. The exact mechanism of esophageal injury is not understood, however, direct thermal injury, acid reflux due to ablation, infection from the esophageal lumen, and ischemic changes in arterioles supplying esophagus are potential culprits [6]. Due to the delayed representation of AE fistulas, direct mechanical insult is less likely the cause of injury. Current theory implicates impaired healing secondary to thermal injury to the esophagus. Heat may damage the endothelial cells of the esophagus or may damage anterior esophageal arteries causing ischemic necrosis of mucosal layers. The delayed presentation of AE fistulas points towards the hypothesis that the esophageal artery ischemia is the primary mechanism of injury. Pre-existing esophagitis due to gastroesophageal reflux disease (GERD) has also been reported to delay the healing process, requiring proton pump inhibitors (PPIs) as prophylactic therapy before ablation [7]. After the necrosis of the esophageal mucosa occurs, the beginning of the fistula formation starts from the esophageal side, extending into the mediastinum, pericardium, and left atrium. This leads to the formation of an AE fistula [8].

Several factors have been implicated in the increased risk of development of AE fistulas. An esophageal injury resulting in ulceration has been the inciting factor resulting in fistula formation. Persistent atrial fibrillation results in left atrial dilation and increased left atrial size. This decreases the distance between the left atrium and the esophagus, resulting in a close connection between them causing esophageal injury [9]. Further studies reported that patients with low body mass had a shorter distance between the left atrium and the esophagus due to decreased tissue interceding them [10]. Wide circumferential ablation of the posterior wall of the left atrium with high power settings can also contribute to the process [11]. It is advised that low energy settings may prevent any adverse outcomes, promoting safety without losing its efficacy. General anesthesia while performing the procedure can also contribute to esophageal ulcerations. The exact mechanism is unknown but peristalsis and decreased swallowing are thought to contribute to the process [12]. All these risk factors are for the development of esophageal ulceration. However, the progression of esophageal injury towards fistula formation is still unknown.

Table 1 provides a summary of the possible mechanisms involved in esophageal fistula formation [13].

**Table 1:** Possible mechanisms of atrial esophageal fistula formation

	Possible Mechanisms of Atrial-esophageal Fistula Formation					
Esophageal dysmotility	General anesthesia					
	Nerve damage during ablation					
Gastroesophageal reflux	Damage to vagal nerve fibers that can impair the esophageal sphincter function and increase reflux, damaging the esophageal mucosa					
Direct mucosal damage	Thermal injury					
	Damage to anterior esophageal arteries causing ischemia and ulceration predisposing atrial-esophageal fistulas					

Prevention strategies may reduce the incidence of esophageal thermal lesions but due to the frequent usage of the procedure, there will still be some cases of AE fistulas reported. The duration, as well as the power of energy application, should be reduced. Frequent monitoring of the temperature inside the esophagus lumen should be done. The position of the esophagus as compared to the left atrium should also be monitored. Patients should be educated about the signs and symptoms of complications due to AE fistulas so that they can present themselves to the hospital as soon as possible without any delay. Despite judicious monitoring and following the above-mentioned protocols, there is still an increased risk of development of esophageal ulceration and subsequent fistula formation [14].

An AE fistula presents three days to five weeks after the initial catheter ablation procedure, although earlier and later onsets have been reported [15]. Signs and symptoms are not specific and consist of fever, malaise, chest discomfort, nausea, hematemesis, odynophagia, melena, and dyspnea [16]. Neurologic defects often secondary to air emboli can result [17]. It is important to note that when an AE fistula is suspected, any instrumentation of the esophagus with endoscopy or transesophageal echocardiogram should be avoided, as blowing of the air inside can cause air emboli and cause severe neurologic manifestations or death [17]. The vagus nerve also courses posterior to the left atrium and thus it can be damaged as well due to ablation. The vagus nerve enters the abdomen and supplies the celiac and lineal plexus, stomach, lesser omentum, and liver. In the gastrointestinal tract, the vagus nerve controls peristalsis, pyloric sphincter relaxation, and gastric antrum motility [18].

When the diagnosis of an AE fistula is suspected, a white blood cell count should be ordered, as it is the sensitive laboratory marker of an AE fistula [19]. Blood cultures may grow a gram-positive organism. In our case, it grew Lactobacillus species. The best diagnostic modality of choice are CT or MRI of the esophagus. Other modalities, such as esophagogram, a transthoracic echocardiogram, can be performed as well. CT and MRI brain is performed to rule out any brain lesions.

**Figure 3:**
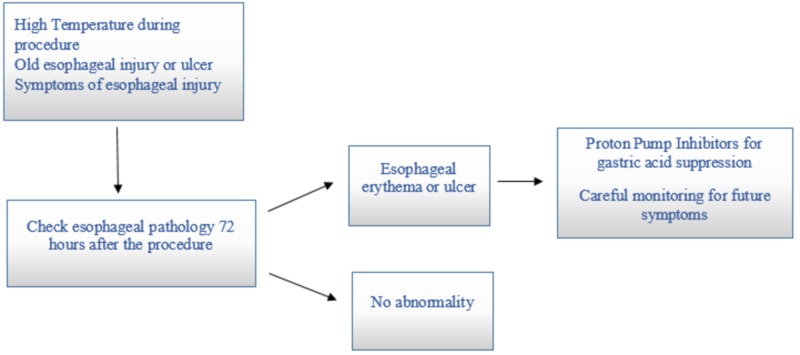
Assessment of esophageal injury post ablation

Various surgical approaches have been suggested for the treatment of AE fistulas. Endoscopic stenting of the esophagus or primary esophageal repair along with placing an omental repair has been used. Early surgical repair is essential because, without it, the mortality rate is 100%. Resection of the necrotic tissue along with patch repair is done. Despite the surgical correction, the mortality rate is still very high, i.e., 41% [20]. Broad-spectrum antibiotics should be added along with gram-negative coverage for enteric organisms but surgical repair is necessary. The following flowchart shows the management workup for patients with esophageal injury post-ablation (Figure 3).

## Conclusions

Despite high monitoring, the risk of AE fistulas and the subsequent mortality remain high. There is an urgent need for newer techniques or modalities to the current treatment to reduce the risk of AE fistulas and the associated mortality. Endoscopy should be avoided once there is suspicion of an AE fistula. Early surgical repair remains the cornerstone of treatment.
